# Conceptualizing socio‐hydrological drought processes: The case of the Maya collapse

**DOI:** 10.1002/2015WR018298

**Published:** 2016-08-16

**Authors:** Linda Kuil, Gemma Carr, Alberto Viglione, Alexia Prskawetz, Günter Blöschl

**Affiliations:** ^1^ Centre for Water Resource Systems, Vienna University of TechnologyViennaAustria; ^2^ Institute of Hydraulic Engineering and Water Resources ManagementVienna University of TechnologyViennaAustria; ^3^ Institute of Statistics and Mathematical Methods in Economics, Vienna University of TechnologyViennaAustria; ^4^ Wittgenstein Centre for Demography and Global Human Capital (IIASA, VID/OEAW,WU)ViennaAustria

**Keywords:** socio‐hydrology, Ancient Maya, drought, vulnerability

## Abstract

With population growth, increasing water demands and climate change the need to understand the current and future pathways to water security is becoming more pressing. To contribute to addressing this challenge, we examine the link between water stress and society through socio‐hydrological modeling. We conceptualize the interactions between an agricultural society with its environment in a stylized way. We apply the model to the case of the ancient Maya, a population that experienced a peak during the Classic Period (AD 600–830) and then declined during the ninth century. The hypothesis that modest drought periods played a major role in the society's collapse is explored. Simulating plausible feedbacks between water and society we show that a modest reduction in rainfall may lead to an 80% population collapse. Population density and crop sensitivity to droughts, however, may play an equally important role. The simulations indicate that construction of reservoirs results in less frequent drought impacts, but if the reservoirs run dry, drought impact may be more severe and the population drop may be larger.

## Key Points

- We present a coupled hydrology‐demography model that simulates plausible feedbacks for the Ancient Maya
- Reservoirs allowed the Maya people to sustain longer economic growth and reach higher population levels
- However, reliance on reservoirs resulted in bigger population drops when major droughts occurred

## 1 Introduction

Many of the water challenges the world is facing today can be better addressed by understanding societal development in the past [*Mithen and Black*, 2011; *Chase et al*., 2014]. There are many approaches for increasing our understanding of societies and ecosystems in the face of water scarcity. They include theories on our position and (changing) relationships with our environment [*Folke*, 2006; *Kallis and Norgaard*, 2010], scarcity indicators to aid policy and management responses [*Falkenmark*, 1989; *Vörösmarty et al*., 2010; *Mekonnen and Hoekstra*, 2011] and models to understand and predict water availability at both the local and global scale [*Davies and Simonovic*, 2011].

In developing models, multiple complementary avenues can be pursued [*Sivapalan and Blöschl*, 2015]. One is to obtain more realism and a better understanding of process relationships for a particular case study through collecting precise data in both space and time and modeling them quantitatively [*An*, 2012; *Bastiaanssen et al*., 2000]. An alternative is to take a step back to find the basic principles underlying the dominant paths observed. In this approach the perspective goes beyond the specifics of individual cases toward the more general patterns [e.g., *Srinivasan et al*., 2012; *Elshafei et al*., 2014].

Following the second approach, this paper presents a stylized dynamic model conceptualizing the interactions between an agricultural society with its water scarce environment thereby drawing from the hydrological, socio‐economic, and vulnerability literatures. The philosophy of conceptualizing both the hydrological and societal processes as part of one socio‐hydrological system, and thus treat social processes as endogenous instead of exogenous, has first been proposed by *Sivapalan et al*. [2012] and is also the aim of the Panta Rhei initiative [*Montanari et al*., 2013]. Similar developments have also occurred in other disciplines such as in the social and ecological sciences [e.g., *Caldas et al*. 2015; *Schlüter et al*., 2012]. Since its introduction, several socio‐hydrological studies have been published [*Di Baldassarre et al*., 2013; *Viglione et al*., 2014; *Pande et al*., 2013; *Elshafei et al*., 2014]. At the long time scales of decades to centuries, potentially slow but continuous adaptation should be accounted for [*Thompson et al*., 2013]. In this respect, taking a historical socio‐hydrological approach and examining ancient societies is of great potential value. The archeological data show that several societies, e.g., the Maya civilization [*Douglas et al*., 2015], the Garamantian population in modern day Libya [*Nikita et al*., 2011] and the Khmer society in modern day Cambodia [*Buckley et al*., 2010], have been able to survive in harsh environments for centuries. What these societies have in common is their ability to shape their environment through reservoirs, tunnels and/or irrigation ditches. This enabled them to exceed the population density based solely on the natural or physical constraints of their environments. While smart water management is no guarantee for success, a society needs to solve basic subsistence problems, no matter how advanced or complex [*Fedick*, 1995]. Also in present times, societies increasingly encounter the limits of available fresh water sources and need to find new solutions [*Vörösmarty et al*., 2010]. Feedback thinking as proposed in socio‐hydrology is thus particularly well suited for understanding water scarcity. For example, *Ohlsson* [2000, p. 213] noted: “the social use of water forms a spiral movement that oscillates between a perceived scarcity of the natural resource water and a perceived scarcity of the social means to overcome the original scarcity, thereby always moving in the direction of increased amounts of social means applied to overcome the water scarcity.” The more inclusive concepts of vulnerability also contain this notion of a balance between exposure and sensitivity on the one side and adaptive capacity on the other [*Füssel and Klein*, 2006; *Polsky et al*., 2007].

The primary goal of this study is to present a model framework that captures the dynamic and relative nature of water scarcity and to elucidate the main drivers that are responsible for society‐water pathways. The second goal is to test whether this framework can simulate representative dynamics of a real world case. The ancient Maya society, being known for its long‐lasting prosperity in an environment in which surface water is not available throughout the year, provides an ideal example. This society that lives in northern Central America, including present day Mexico, Belize and Guatemala, experienced a peak in population at the end of the Classic period (AD 600 to 830), after which a rapid depopulation followed [*Aimers*, 2007]. Around AD 1500 the remaining Maya people were conquered by the Spanish [*Graham et al*., 1989]. The expansion of the Maya civilization and the associated depopulation has been attributed to many causes, such as soil erosion, climate change, deforestation, disease, class conflict, inter‐site warfare, and a change of trade routes [*Aimers*, 2007]. Archaeological evidence shows that the area did not uniformly depopulate, but points to regional and site‐by‐site variability, which suggests there is no simple narrative [*Aimers*, 2007, 2013]. Of the multiple causes the possible role of climate, especially drought, has been a recurrent one. Attempts to understand and quantify the link between climate and the Maya civilization are based on analyses of Maya records of the Chilam Balams and early Colonial histories, on establishing theories that link global climate patterns to local phenomena [*Gunn et al*., 2002a] and on isotope analysis of sediment cores, stalagmites or plant wax [*Hodell et al*., 2005; *Medina‐Elizalde et al*., 2010; *Douglas et al*., 2015; *Kennett et al*., 2012]. While in earlier studies the hypothesis of a mega‐drought lasting several decades to a century was put forward [*Hodell et al*., 1995; *Curtis et al*., 1996], more recent studies indicate the occurrence of multiple droughts [*Medina‐Elizalde and Rohling*, 2012; *Douglas et al*., 2015; *Kennett et al*., 2012]. The relationship between climate variability, local environmental conditions and associated land and water management practices is subject to debate [*Chase and Scarborough*, 2014; *Dunning et al*., 2012; *Ford*, 1991; *Heckbert*, 2013; *Lucero*, 2002]. Therefore, our third goal is to contribute to the debate on the role of climate in the rise and fall of the Maya society by testing if the prevailing drought hypothesis is consistent with the model framework put forward here.

## 2 Model Conceptualization

The ultimate aim of the proposed model is to investigate the feedbacks between water stress and society. We therefore chose food as a central element of the model. Even today, 70% of the freshwater resources globally are used for food production [*FAO*, 2015]. For our study, we imagine ourselves at the origin of a settlement and conceptualize the processes as follows: a number of people arrive in an area and decide to stay. In order to fulfill their basic needs they start to cultivate the area around them for food thereby removing the natural vegetation. Per area food production depends on crop characteristics and on the amount of precipitation that falls. Having enough food results in the growth of the population which in turn requires more land to be cultivated in order to further raise food production, which allows further population growth. There are two ways in which this feedback can be interrupted, i.e., either from within the system or through events occurring outside of the system (or in effect it is an interplay of both). Firstly, considering that land is limited, the dominant, average amount of precipitation into the system is limited and that there is a given set of knowhow and skills to produce food, population cannot grow indefinitely. Thus, when food supply can no longer be increased despite food demand remaining high, the community becomes vulnerable. Secondly, it could happen that precipitation deviates from what is considered normal and the community faces reduced harvests as a consequence of drought. Here the community can become vulnerable, too. In absence of any adaptive measures, the reduction of population will occur. This could either occur through reduced births, increased mortality or forced emigration. While there are multiple ways to improve water security, we consider only the structural measures of reservoir building. An increase in the society's vulnerability motivates them to act and reservoirs are constructed. The reservoir affects the hydrological system by capturing more water, allowing for an increase in the food production per area and a restoration between food supply and food demand, allowing renewed population growth. This feedback continues until the society realizes that building more reservoirs is no longer resulting in increased water supply, a process that is captured by the society's social knowledge or memory.

**Figure 1:**
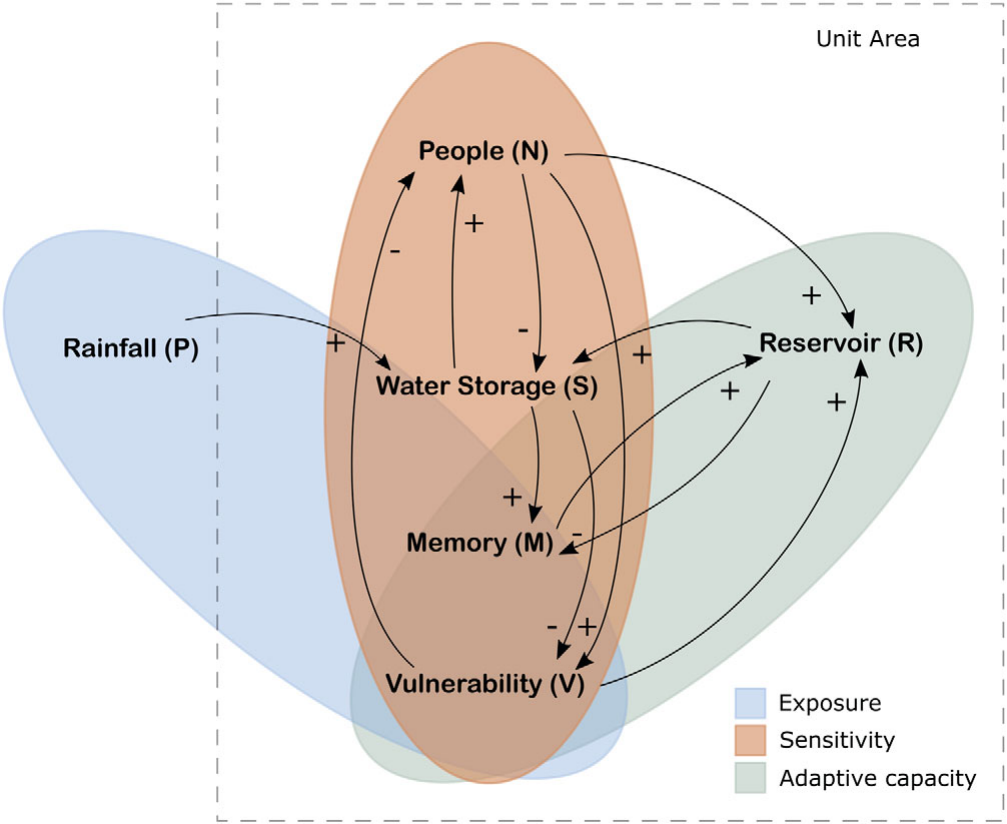
Flow diagram showing the state variables of the model and how they interact. When *P* increases, water storage *S* increases (+), resulting in a higher yield and population *N* can grow (+). The use of water by the society leads in turn to a decrease in storage (−). If population increases this could potentially lead to a higher vulnerability *V* of the system (+), although increased water storage *S* counteracts this (−). Increased vulnerability *V* motivates society to construct reservoirs *R* (+), provided that there are enough people (labor) to do so (+). More reservoirs allow increased water storage (+). If storage is high relative to the storage capacity, memory *M* is high and reservoir construction *R* continues, but by creating more storage, *M* decreases (−). Lastly, when the system is vulnerable (i.e., close to 1) and a threshold is reached, population *N* is impacted negatively (−).

In order to conceptualize the above dynamics, we build a stylized mathematical model that captures the key state variables of this socio‐hydrological system and accounts for feedbacks. We assume a continuous state space in which the dynamic evolution of the state variables is captured by nonlinear differential equations. Figure 1 shows the state variables and feedbacks of the model, namely water storage (*S*), population density (*N*), reservoir storage (*R*), memory (*M*) and vulnerability (*V*). The model is driven by precipitation (*P*) that includes drought events.

The choice for differential equations is motivated by their suitability for understanding general behavior of complex environments, as feedback mechanisms can be incorporated and valuable insights can be obtained in cases for which data availability is low [*Brown*, 2007]. The approach is also suitable because we are focusing on two‐way interactions, and the model framework needs to be capable of dealing with tangible and intangible feedbacks for which data availability is not guaranteed.

Before describing the equations in detail, it is important to note that our model is bounded, meaning that the hydrological and the social dynamics are considered within a unit area. Processes such as the inflow of water from upstream areas, but also trade between communities is therefore not included. Furthermore, the unit of water [L] is used as much as possible throughout the model. This allows us to focus on the essential interactions important for the hydrological system instead of diving into the complexity of, for example, how to price water [*Savenije and Van Der Zaag*, 2002; *Brouwer and Hofkes*, 2008].

### 2.1 Hydrology

The hydrology is modeled as a simple water balance equation for a unit area (see equation (1)), where *S* denotes the total water storage per unit area [L] and is composed of the soil moisture storage, *S_S_* [L], and the water stored in the reservoirs, *S_R_* [L]:

(1) $$\begin{array}{l} \frac{dS}{dt}=P-ET_{crops}-ET_{native}-E_{R}-Q\\ =P-\alpha_{H}\frac{S_{S}}{\phi_{H}}\text{min}\left[\frac{\mu_{N}N}{K},1\right]\\ \;\;-\beta_{H}\frac{S_{S}}{\phi_{H}}\left(1-\text{min}\left[\frac{\mu_{N}N}{K},1\right]\right)-\gamma_{H}*S_{R}\\ \;\;-P\frac{1}{1+e^{-\epsilon_{H}*\left(S_{S}/\phi_{H}-\delta_{H}\right)}}\frac{1}{1+e^{-\eta_{H}\left(S/(\phi_{H}+R)-\zeta_{H}\right)}} \end{array}$$

**Figure 2:**
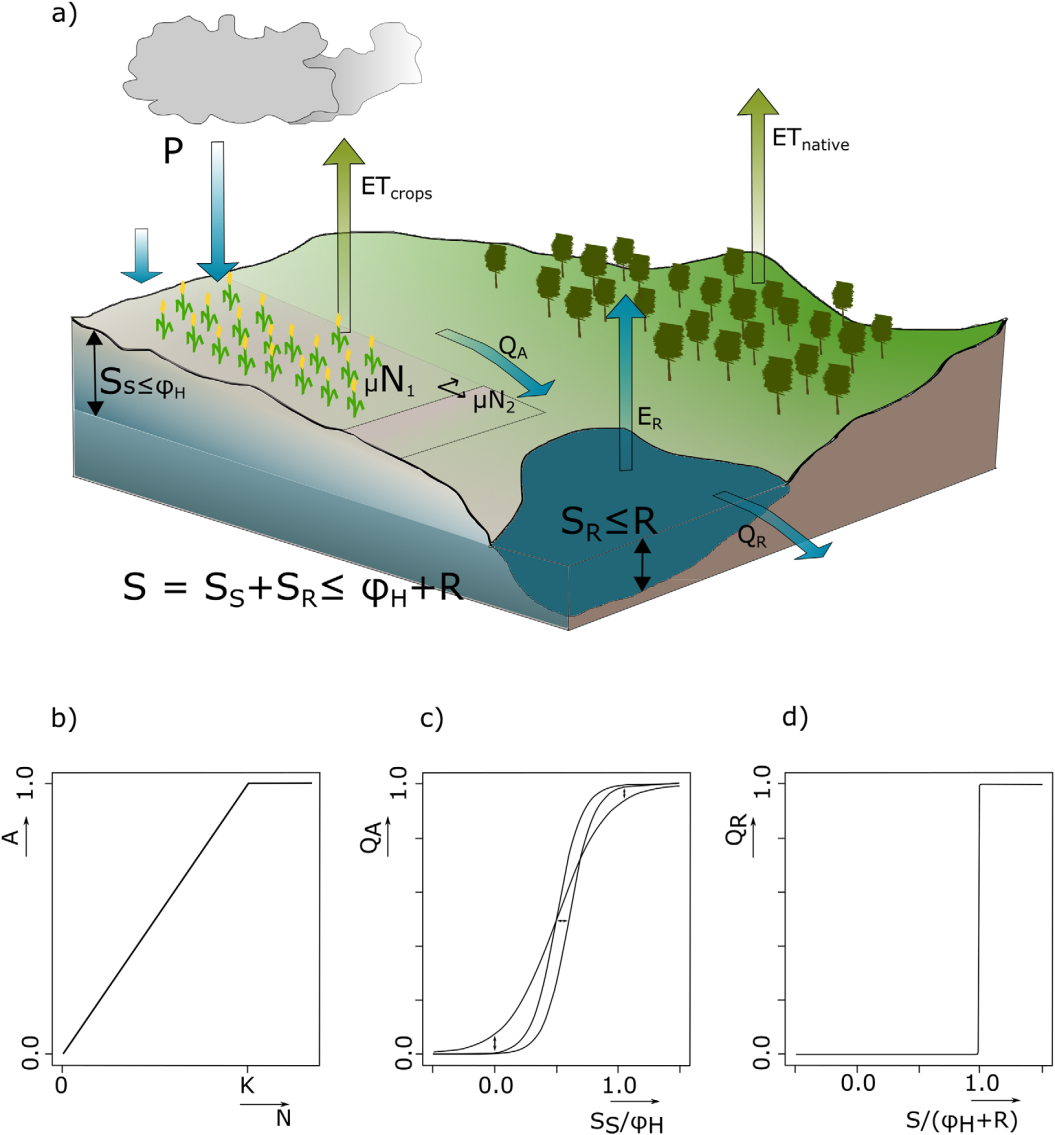
Illustration of the dynamics captured by the hydrology equation (equation (1)). (a) The in‐ and outflows of the unit area are precipitation (*P*), evapotranspiration from the cropland (*ET_crops_*) and area with native vegetation (*ET_native_*), evaporation from the reservoir (*E_R_*), runoff from the area (*Q_A_*) and spillover from the reservoir (*Q_R_*). *μ_N_N* represents the fraction of land that is cultivated per unit area; when population increases, e.g., from *N*_1_ to *N*_2_, the cultivated land increases from *μ N*_1_ to *μ N*_2_. (b) Relation between Area (*A*) and population (*N*). (c) Runoff (*Q_A_*) as a function of soil saturation $\left(S_{S}/\phi_{H}\right)$. (d) Spillover as a function of the ratio of actual water storage per area to the total water storage capacity available $S/(\phi_{H}+R)$.

*S* is a function of precipitation (*P*), evapotranspiration from the cultivated area (*ET_crops_*), evapotranspiration from the natural vegetated area (*ET_native_*), evaporation from the reservoir (*E_R_*), and Q, which is the sum of runoff (*Q_A_*) and spillovers from the reservoir (*Q_R_*). For simplicity it is assumed that the reservoir is located at the outlet of the area. Soil moisture storage *S_S_* and water storage in the reservoir *S_R_* are limited by field capacity, $\phi_{H}$ [L], and reservoir capacity, *R* [L], respectively. Field capacity is here defined as the water content of the soil after drainage has stopped, which is an ideal situation for crop growth [*FAO*, 1985]. *R* is a variable of the model. The simplifying assumption is made that water moves from the reservoir to the soil as long as the soil is below field capacity, i.e., irrigation takes places in order to keep the water content at an optimum. Only when the soil moisture storage is larger than field capacity, *S* > $\phi_{H}$ (and *R* > 0), water is stored in the reservoirs, *S_R_* > 0. Hence, *S_S_* is calculated as $\text{min}[S,\phi_{H}]$, while *S_R_* is calculated as the $\text{max}[S-\phi_{H},0]$. An overview of the in‐ and outflows is presented in Figure 2a.

Precipitation, *P* [L T^–1^], is a flux and exogenous to the model. Crop evapotranspiration, i.e., *ET_crops_*, is a function of the maximum evapotranspiration rate possible given by *α_H_* [L T^–1^], water stress represented by the ratio of actual soil moisture *S_S_* over field capacity $\phi_{H}$ (see for a similar assumption the water balance model of *Schaake et al*. [1996]) and the fraction of cultivated land. With respect to the latter, it is assumed, that when the total unit area is cultivated, the number of people that can live off the land is *K* [people] or the carrying capacity of the land. People cultivate the land proportionally to the number of people, represented by *N*. Hence, the fraction of land that is cultivated can be expressed by the ratio of actual people to carrying capacity, where the size of the land that is actually cultivated per person can be adjusted by *μ_N_*, see also Figure 2b. We postulate that *K* is not constant in this model framework. Assuming that, in theory, all the water stored in the soil during a time period can be used for crop growth and introducing a parameter *γ_N_* that represents a measure of the water requirement per capita per time that is used to fulfill a food requirement per capita per time [L T^–1^ Person^–1^], K can be expressed as the average water availability per time $\bar{S_{S}}=(S_{S}+S_{S(t-c)})/2$ divided by the average water need per person per time, i.e., $K=(\bar{S_{S}}/\gamma_{N})$. Symbol c represents the lagtime to estimate the average. Consequently, during the years when there is little water, the carrying capacity of the land is reduced.

Evapotranspiration from the native vegetation or *ET_native_* is, similarly to *ET_crops_*, a function of a maximum evapotranspiration rate given by β*_H_*, water stress experienced by the plants and a function of area, where the latter is calculated as 1 minus the cultivated area. The evaporation from the reservoir is calculated as the evaporation rate *γ_H_* [T^–1^] multiplied by the amount of water in the reservoir. Runoff and/or spillover from the reservoir are represented by the last term of equation (1). The first logistic function in the denominator ensures that, if soil moisture *S_S_* equals field capacity $\phi_{H}$, any additional precipitation will run off and its output is *P*. However, even if the soil is not saturated runoff occurs. The amount of runoff, similar to the run‐off coefficient, can be set by the dimensionless parameters ${ɛ}_{H}$ and *δ_H_*. The principle is illustrated in Figure 2c. The second logistic function governs reservoir storage. For simplicity, it is assumed, that the society is able to capture all runoff from the fields, although this can be easily changed by adding an additional efficiency parameter. Depending on the parameter choice of *η_H_* and *ζ_H_*, the reservoir can release water even though it is not full. Currently, the parameters are set to represent a step function; there is only spillover from the reservoir when it is full and $S/(\phi_{H}+R)$ equals 1, see Figure 2d.

### 2.2 Population

Population dynamics are represented by *N* and measured as the number of people per unit area [persons *L* ^–2^], see equation (2a). We assume Malthusian population dynamics [*Malthus*, 1798]; the society is able to grow as long as it has enough resources and experiences a decline when the resources fall below a minimum level or subsistence level. While industrialized countries, after having experienced a post‐Malthusian regime, are characterized by a modern growth regime, the theory of Malthus is generally accepted to describe the socio‐economic situation before industrialization took place [*Kögel and Prskawetz*, 2001; *Galor and Weil*, 2000; *Hansen and Prescott*, 2002].

The equation governing the population dynamics is:

(2a) $$\frac{dN}{dt}=(b-d)N$$

where

$$\begin{array}{cc} & b=\alpha_{N}+\frac{\theta_{N}}{1+e^{-\delta_{N}\left(FA-\beta_{N}\right)}}\\ & d=\left(\alpha_{N}+\frac{\epsilon_{N}}{1+e^{-\delta_{N}\left(\beta_{N}-FA\right)}}\right)\left(1+\zeta_{N}V^{\eta_{N}}\right) \end{array}$$

where b is the per capita birth rate and d the per capita death rate. Both rates vary depending on food availability *FA*, which is calculated as the ratio between the food produced (yield) and the food need of the community (see equation (2a)). The variable *V* is the measure of vulnerability.

**Figure 3:**
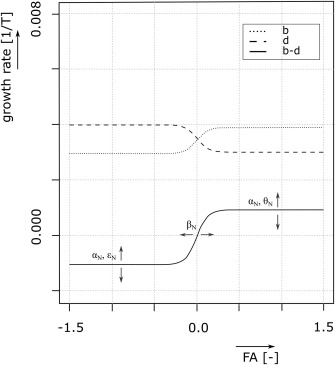
The response of birth and mortality rates to food availability. For a preindustrial society the mortality rate will still be high due to exogenous circumstances to the model, e.g., poor medicine availability and hygienic conditions, even though food surplus occurs.

Birth rate increases and mortality decreases with food availability, Figure 3. *θ_N_* [T^–1^] determines how strongly the birth rate responds to changes in food availability. Together with *α_N_* [T^–1^] the overall birth rate can be set, but note that *α_N_*, in combination with $\epsilon_{N}$ [T^–1^], is also determining the mortality rate. *α_N_* is considered a parameter that reflects the type of development, i.e., a preindustrial or industrial society. Both birth rate and mortality rate approach a constant limit for large food availability due to factors external to the model, such as poor hygiene conditions [*Kirk*, 1996]. For simplicity, it is assumed that the birth rate follows the mortality and the same parameter *α_N_* is used in both the mortality and the birth rate. In combination with *θ_N_*, *α_N_* can be considered as the overall outcome of society's fertility preferences.

In the case of a long‐term food shortage, the vulnerability of the community increases (see equation (3)) and an additional feedback occurs. The parameters controlling the moment and the extent of this impact are a multiplication factor *ζ_N_* and a power factor *η_N_* that are both dimensionless. A similar formulation has been used in *Gragnani et al*. [1998] to model the link between environmental pollution and population.

The last two parameters are *β_N_* and *δ_N_*. Together they give shape to the transition period when the society moves from a food surplus to a food deficit situation. Parameter *β_N_* is the inflection point and the midst of the transition from dynamics associated with food surplus to dynamics characterized by food deficit. Parameter *δ_N_* determines the speed and smoothness of the regime shift, i.e., how fast does the society adjust its birth and mortality rate when food becomes scarce. For simplicity, it is assumed that *δ_N_* and *β_N_* are the same for the mortality rate and the birth rate. In reality the timing and magnitude of these responses will be different. Overall, in line with Malthus' theory, the population equation allows for both a preventing check and a positive check to occur. The preventive check occurs when society adjusts its net growth rate in response to food shortage. This is a gradual adaptation that may or may not occur timely. If the adjustment is too slow, and the population size remains above its carrying capacity too long, the society may become vulnerable leading to a positive check, which could be famine and/or emigration.

#### 2.2.1 Food Availability Indicator

The food availability indicator is defined here as the ratio between the food that is produced and the food that is demanded by the society. The production or yield [L T^–1^] is based on the FAO water production function and a function of water stress experienced by the crop [*Steduto et al*., 2012]. The function accounts for the fact that yield response to water varies according to crop type and growth stage in which water stress occurrs and is expressed as: $\left(1-\frac{Y_{a}}{Y_{x}}\right)=K_{y}\left(1-\frac{ET_{a}}{ET_{x}}\right)$, where *Y_x_* and *Y_a_* are the maximum and actual yields, *ET_x_* and *ET_a_* are the maximum and actual evapotranspiration, and *K_y_* is a yield response factor. Rewriting the above equation to obtain actual yield as a function of actual and maximum evapotranspiration, realizing that maximum yield is equal to evapotranspiration as all is written in [L T^–1^] and writing the yield response factor as *θ_H_* gives equation ((2a)). Parameter *θ_H_* = 1, if yield is linearly related to water, > 1 if the crop response is very sensitive to water deficit and results in proportionally larger yield reductions when water use is reduced, and < 1 if the crop is relatively drought tolerant and may recover. Food demand is calculated as the water requirement per person, *γ_N_* [L T^–1^ Person^–1^], multiplied with number of people *N*. If reservoirs are built an additional temporarily cost is posed on society, equal to $\alpha_{R}NMV(1-V)$ (see also equation (4)). It is assumed that all of the food produced is available as food supply:

(2b) $$FA=\frac{-\alpha_{H}\left(\left(\left(1-\frac{S_{S}}{\phi_{H}}\right)\theta_{H}\right)-1\right)\text{min}\left[1,\frac{\mu_{N}N}{K}\right]}{\gamma_{N}N+\alpha_{R}NMV(1-V)}-1$$

### 2.3 Vulnerability of the Community

The variable vulnerability (V) represents a measure of the state of vulnerability of the society and is represented by the following equation:

(3) $$\frac{dV}{dt}=-FA(V-\text{min}\left[0,FA\right])(1-V)$$

where *V* is a measure of vulnerability and depends on food availability (FA) (see equation ((2a))). The value of *V* varies between 0 and 1, where 0 represents a non vulnerable community (that can easily cope with a sudden change in precipitation) and 1 a vulnerable community (that experiences a shock and possibly collapse, for the same drop in precipitation). From the equation it becomes apparent that food availability drives the vulnerability status. Indirectly, however, food availability is the result of the amount of water input to the system, (artificial) storage capacity and the number of people living in the area. Vulnerability is in effect the interplay of the systems exposure, sensitivity and adaptability, or the dynamic outcome of the (initial) resources at hand provided by nature, the demand created by society and the ability of humans to effectively enlarge the resource base and to use it efficiently.

The use of the term vulnerability follows the biophysical line of thinking within the vulnerability literature [*Turner et al*., 2003; *Eakin and Luers*, 2006], for which a risk/hazard, i.e., drought, is central to the model. The IPCC definition applies, i.e., vulnerability is “the degree to which a system is susceptible to, or unable to cope with, adverse effects of climate change, including climate variability and extremes (drought). Vulnerability is a function of the character, magnitude, and rate of climate variation to which a system is exposed, its sensitivity, and its adaptive capacity” [*Füssel and Klein*, 2006, p. 306]. Vulnerability is variable in the model, which addresses the important point in the literature that vulnerability is not a static, but a dynamic attribute [*Downing et al*., 2005].

While food availability can change quickly from one time period to the next, vulnerability changes more slowly. Hence, when long‐term food shortage occurs, the community gradually moves from a nonvulnerable to a vulnerable state. In combination with the population parameters *ζ_N_* and *η_N_*, vulnerability aims to capture the notion that an increased vulnerability can cause a shock to the population. It is also possible to move back to a nonvulnerable state and avoid a shock, if the population is able to respond timely. Because the basic assumption in the model is a self‐sufficient community, the vulnerability of the society here completely depends on what is produced within the system.

### 2.4 Supply Management

The equation governing reservoir building is:

(4) $$\frac{dR}{dt}=\alpha_{R}NMV(1-V)-\left(\beta_{R}/N\right)R$$

where *R* is reservoir storage per unit area [L], *N* is population, *V* is vulnerability, *M* is memory, *α_R_* is a building rate per person [L T^–1^ person^–1^] and *β_R_* is a depreciation rate [T^–1^ person^–1^].

We refer to reservoirs in this context as structures that are built off‐stream and capture water through harvesting rainwater, as is nowadays still practiced in arid or semiarid regions to provide water for agriculture and households [*Boers and Ben‐Asher*, 1982]. First, we aim to capture the incentive of the society to act in response to its vulnerability, i.e., a crisis creates a window of opportunity [*Pelling and Dill*, 2009; *Birkmann et al*., 2010]. The incentive to invest in solutions is higher in the face of a crisis and its aftermath. The society is assumed to build if 0 < *V* < 1, but if *V* = 1 the construction is halted because the society is in the midst of its crisis. Second, to account for the fact that labor is needed to construct artificial storage, the building rate is assumed proportional to the population density (*N*). *Boserup* [2005] first linked densely populated agricultural communities to the development of more intensive forms of agriculture, as she argued that the willingness to invest additional labor increases when the need for increased food production is higher. Third, to account for the fact that additional reservoir capacity is only useful when there is a chance of being filled, the community builds up social knowledge or memory by observing the system. Memory is given by the ratio of actual storage to storage capacity and consequently, if the gap between actual storage and reservoir capacity becomes large (*M* is small), the community finds itself having enough storage and the building rate slows down. Finally, it is assumed that the constructed storage degrades or silts up with rate *β_R_*∕*N* if not maintained, depending on the amount of people living in the area.

### 2.5 Memory

Memory, as used here, represents local knowledge on the average water availability in the area in relation to the overall storage capacity. The equation governing the memory of society:

(5) $$\frac{dM}{dt}=\frac{S}{\phi_{H}+R}-\gamma_{R}*M$$

where *M* is Memory, *S* is actual storage in the area [L], and *R* + $\phi_{H}$, which represent reservoir capacity [L] and field capacity [L] respectively, make up the total storage capacity of the area. The parameter *γ_R_* represents the loss rate of memory [T^–1^].

One would expect local knowledge on water availability to exist, especially if knowledge on rainfall patterns and water availability is crucial for livelihood. Farmers in Burkino Faso, for example, have a stake in the nature of rainfall, so all community members eventually acquire perceptions and expectations regarding the season [*Roncoli et al*., 2002]. *Barrera‐Bassols and Toledo* [2005] found that knowledge of water availability still plays an important role in the life of contemporary Yucatec Maya farming communities. Here, we assume that the society has no predictive foresight and only base their knowledge on their past observations. The formulation is similar to *Di Baldassarre et al*. [2013] and *Viglione et al*. [2014] in case of flood events, although in their conceptualization memory is only renewed in case of a flood event, while the renewal here is continuous.

## 3 The Socio‐Hydrological System of the Ancient Maya

The socio‐hydrological model is now applied to the Maya civilization. In this section, a short description of the Maya‐civilization is presented, focusing on those elements that are important for the socio‐hydrological framework. It gives a brief overview of the hydrological system, the Maya economy, observed demographics and land and water management practices.

### 3.1 Hydrological System

**Figure 4:**
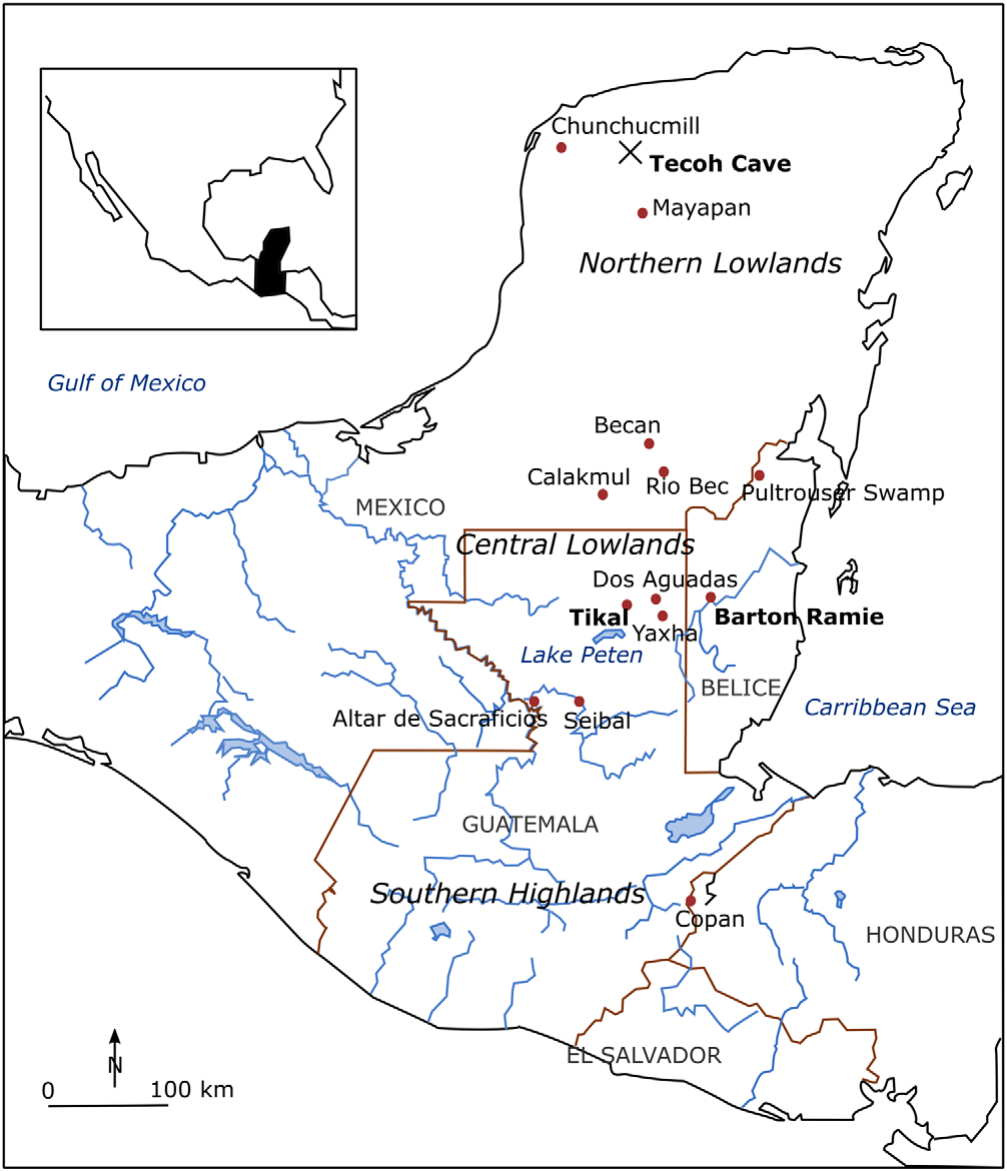
Map showing the most important Maya sites and a division between low‐ and highlands. The region comprises present day Mexico, Guatemala and Belize (and to a lesser extent Honduras and El Salvador). Figure adapted from *Santley et al*. [1986].

The climate of the Maya was characterized by mean annual rainfall ranging from 500 mm in the northwest to 2500 mm in the south, a substantial year to year variation and a pronounced dry and wet season [*Dunning et al*., 1998]. The wet season accounts for 80% of the annual rainfall and usually lasts from May to October (Aleman and Garcia, 1974 in *Hunt and Elliott* [2005]). The lowlands form a low lying area with elevations below 500 m (See Figure 4). The area is karstic (Tertiary Period) with older limestones found in the southern or central Maya lowlands that are characterized by well developed drainage and more relief [*Bauer‐Gottwein et al*., 2011]. Original vegetation of the lowlands consisted of deciduous dry tropical forest and scrub in the north and seasonal to evergreen moist tropical forest in the south, although a change to a more herbaceous and secondary forest is observed during 4000 and 3000 year BC at the same time that maize pollen appeared [*Dunning et al*., 1998]. Due to the combination of karstic geology and elevation, water can only be found just below the surface (via sinkholes or cenotes) in the north, while in the central lowlands, the groundwater table was generally too deep to be accessed [*Dunning et al*., 1998]. Some areas, such as the Three River area and Rio Hondo area, were characterized by rivers and seasonal wetlands, which provided both an opportunity in times of water need but also a limitation due to their inundation and inhabitability.

### 3.2 Demographics

**Figure 5:**
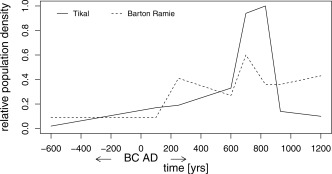
Graph showing estimated population dynamics for a major centre, i.e., Tikal, and a minor centre, i.e., Barton Ramie, for the period 600 BC to AD 1200. The maximum population density thought to have lived in Tikal is set to one, all other data is relative to this number (data from *Culbert* [1988]).

The large variations in landscape features suggests, that Maya communities faced different living conditions depending on where they settled. The availability of groundwater, seasonal wetlands, rivers or streams, the presence or absence of a sufficiently developed soil layer and the kind of agricultural techniques the Maya's had access to determined whether a particular area was cultivated as well as the quantity harvested [*Dunning et al*., 1998]. The agricultural (carrying capacity) approach has been used to estimate population densities based on information and/or assumptions on the physical geography of the regions [*Turner*, 1976]. A second method commonly used to estimate population densities is a house site approach, whereby houses are counted and assumptions are made on the duration of occupation and average family size. Available population records for the region are limited to specific sites and representative for a certain time period. The estimates found in *Culbert* [1988] (and to some extent *Santley et al*. [1986]) are subject to numerous assumptions and associated uncertainty. They include however, an overview of surveyed areas, relative population densities through time and estimates of maximum population densities. Among these are Altar de Sacrificios, Barton Ramie, Tikal, Tikal‐Yaxhá, Becán and areas (Macanché, Salpéten) around Lake Peten (mostly in the Central Lowlands area––see Figure 4). For those locations for which Middle Preclassic occupation has been observed, recorded densities were less than 10% of the maximum reported figures. Subsequently, densities of less than 25% have been reported during the Late Preclassic, after which variation in densities is observed for the Early Classic (< 10–55% of maxima). During the Late Classic, rapid population growth occurred and most of the sites reached their population maxima, followed by a decline during the Terminal Classic. While the majority of the large sites show this pattern, not all of the smaller sites followed this decline. Indications are that populations lasted throughout the Terminal Classic in Barton Ramie and throughout Belize, although only for Barton Ramie quantitative data exist. Around Tikal populations never recovered. Figure 5 shows the demographics for a major city center and for a minor city center. The above mentioned periods in Maya history correspond to the following dates: Early Preclassic (2000–1000 B.C.), Middle Preclassic (1000–300 B.C.), Late Preclassic (300 B.C.–A.D. 250), Early Classic (A.D. 250–600), Late Classic (A.D. 600–830), Terminal Classic (A.D. 830–930), Postclassic (A.D. 930–1500) [*Culbert*, 1988].

*Culbert* [1988] provides an estimate of the number of people that would have lived in a particular area. Based on the number of structures counted in the various studies, assuming a (conservative) occupation of 50% and an average household size of 5.6 people, he estimated (based on data of Tikal) that during its height around 500 people km^–2^ or more lived in the major centers, while smaller sites had a density of 300 people km^–2^. He furthermore estimated that densities of the rural area and/or minor centers would be similar in number to the outer ring of Tikal, i.e., 168 people km^–2^.

### 3.3 Economy

The proposition that the Maya civilization was dependent on subsistence agriculture is generally well accepted. Ambuigity exists regarding the extent to which different subsistence techniques like swidden agriculture or double cropping were used (see also the next section) and also with regards to the occurrence of trade. Various arguments can be found in the literature both arguing for and against a more prominent role of trade and markets. Some authors suggest that centers, such as Chunchucmil [*Dahlin et al*., 2007] or Wild Cane Cay [*McKillop*, 1996] are strategically located and could have functioned as trade centers. *McKillop* [1996] found obsidian, gold, exotic pottery and copper at different settlements along the coast. Obsidian was more widely distributed than the other products mentioned, indicating that not all of these exotics were available for commoners, but restricted to elites in some cases. Based on geochemical analyses of soils in Chunchucmil [*Dahlin et al*., 2007] and Caracol [*Chase and Chase*, 2014], the authors identified places that are indicative of increased human activity and vending of craft and potentially food items. However, in general, as food is organic and perishable it is very hard to find traces in the archaeological records [*Chase and Chase*, 2014]. Furthermore, evidence for trade is mainly related to the Post‐Classic period and extrapolation into earlier periods, such as the Classic period, is extremely difficult [*Dahlin et al*., 2007]. In absence of easy transport routes, such as rivers, scholars have argued that the distance at which transport energy does not exceed the energy of the food being transported could be indicative of a spatial scale of trade. *Turner* [2000, p. 252] argues that the distance between the northern Belize zone of wetland fields and central Peten (focusing on Tikal) is, with a distance of 150 km, probably at the border of transport efficiency. These considerations lead some to conclude that trade might have played a larger role in sites that were relatively easily accessible, i.e., coastal communities or communities near rivers or lakes [*Dunning et al*., 2012]. It is also thought that “useful” subsistence goods were mainly traded locally, while long‐distance trade routes were set up for the import of “functional” luxurious goods for the elite [*McKillop*, 1995; *Tourtellot and Sabloff*, 1972].

### 3.4 Land and Water Management

It has long been thought that the only type of agriculture that the Maya practiced was swidden farming, where after forests have been burned, the plots are used for a couple of years and then left fallow for as long as needed in order to restore soil quality [*Cowgill*, 1962; *Turner*, 1974]. The discovery of forms of intensive cultivation and the contradicting outcomes of population densities estimated by the agricultural carrying capacity versus the house count approach (the former giving much lower densities than the latter) led to the belief that more intense forms of agriculture were practiced [*Turner*, 1974]. Modifications of the landscape have been found around the centres of Tikal, Caracol, Pulltrouser Swamp, Blue Creek, Calakmul and Edzna. In case of Tikal, remains of a series of (man‐made) reservoirs has been discovered, forming a cascade through the city, ending in a bajo––an internally drained, seasonally inundated depression [*Dunning et al*., 1998; *Scarborough et al*., 2012]. Furthermore, research has revealed that at least part of the nearby bajos show signs of human labour and also remnants of weirs and dams have been discovered [*Dunning et al*., 2002]. To what extent the Maya modified bajos remains subject to debate. For the center of Caracol, references to the presence of terraces go back to the 1920s [*Healy et al*., 1983]. Recent Lidar data have confirmed this, showing large scale landscape modifications in that area [*Chase et al*., 2011]. With the use of terraces, additional land could be cultivated as soil erosion was slowed down and the water retention capacity was increased. Evidence for wetland use is found in northern Belize, around the centres of Pulltrouser swamp and Blue Creek, ca. 40 km south of Pulltrouser swamp) [*Luzzadder‐Beach et al*., 2012]. While the former location indicates modifications related to the use of natural hammocks within the wetland, the latter shows evidence of the existence of canals to both drain and feed the system as needed. Furthermore, research around the site of Calakmul indicates the use of wetlands and the formation of an extensive canal system including reservoirs in the Edzna valley to the west of Calakmul basin [*Gunn et al*., 2002b].

## 4 Results: The Collapse of the Maya

For the model setup we chose parameters that are consistent with our knowledge of the socio‐hydrological system of the Maya with the aim of gaining insight into the circumstances under which a collapse would have been plausible in a major population center such as Tikal.

### 4.1 Model Setup

#### 4.1.1 Precipitation and Initial Values

The precipitation series (from AD 487 to 1000) is based on the data of *Medina‐Elizalde et al*. [2010], who derived an annual rainfall record from a stalagmite taken from Tecoh cave that is located in the north east of the Northern Lowlands (see Figure 4). Mean annual rainfall is much lower (i.e., 1120 mm versus 1800 mm [*Chase et al*., 2014]) at this location than at Tikal, so the data of *Medina‐Elizalde et al*. [2010] have been scaled by a factor of 1.607. In order to create monthly rain data and a distinct rain season in which around 80% of the rain falls within six months, the annual precipitation values have been downscaled to monthly values and by applying a sinus function with an amplitude of 0.9 times the monthly mean.

The population *N* has been set to 20 persons per km^2^ in the year 487, in line with the relative population density in the Early Classic as mentioned by *Culbert* [1988]. Initial (soil moisture) storage *S* was set to 160 mm (somewhat below field capacity). Reservoir capacity *R*, vulnerability *V* and memory *M* were all set to 0.

#### 4.1.2 Parameters

Maize was an important staple crop of the Mayans [*Cowgill*, 1962]. It needs 500 to 800 mm during its three to five month growing season in order to mature [*FAO*, 2013]. Multiple harvests are reported to have been possible [*Chase et al*., 2010; *Wilken*, 1971]. In the model we have set *α_H_* to a value of 200 mm per month. This implies that the Maya could, assuming water is present during the entire year, cultivate up to three harvests per year. In practice, the system is water limited and so most of production in the model will take place during the rainy season resulting in a lower number of harvests per year. The value of *β_H_* is set equal to *α_H_* for simplicity. An estimate of $\phi_{H}$ can be obtained from the average soil depth and the average root depth of the plant being cultivated, and by multiplying this with the soil's porosity to obtain the water content. Estimates of topsoil depth in the Tikal region vary between 15 and more than 100 cm, with an average depth of 51 cm [*Burnett et al*., 2012]. A porosity of 0.25 (min. sand) to 0.7 (max. clay) gives a maximum possible water content of 125 to 350 mm out of a total of 510 mm. Assuming a sand/clay mixture in accordance with the observations of *Burnett et al*. [2012], $\phi_{H}$ has been set to 195 mm. The value of 0.04 of *γ_N_* implies that, if no further inflows or outflows occur, the reservoir loses almost 40% of its water annually due to evaporation. While no values could be found for the Maya region, *Craig et al*. [2005] estimate that small dams in Queensland, Australia, which is also characterized by a tropical wet and dry climate, can lose up to 40% of their water when continuously filled. A value of 0.04 therefore seems a reasonable choice. Both *δ_H_* and ${ɛ}_{H}$, set to respectively 10 and 0.5, determine the run‐off coefficient as a function of soil moisture content and roughly conform to stylized facts [*FAO*, 1991]. Parameters *η_H_* and *ζ_H_* determine the overflow from the reservoir and have been set such that they mimic a step function, i.e., 500 and 0.98. The yield response factor for maize is estimated at 1.25 for the total growing period, meaning that the crop is sensitive to water stress [*FAO*, 2013]. The parameter *θ_H_* is therefore set to 1.25.

Annual crude birth and death rates of 30–40 persons per 1000 were common before the demographic transition [*Khan*, 2008]. Parameter *α_N_* is therefore set to 0.003 per month or 0.036 per year. Annual growth rates have been reported by *Santley* [1990] in [*Lutz et al*., 2000] and vary between 0.0015 and 0.015 in the Early to Late Classic period. As birth and death rates are variable in the model we chose rather high end values to obtain a maximum growth rate of 0.011 per year, i.e., *θ_N_* = 0.00095 when there is food surplus, and a minimum annual growth rate of −0.014 per year in case of food shortage, i.e., $\epsilon_{N}$ = 0.0012 per month. It is thus assumed that the mortality response to food shortage is stronger than the response of the birth rate to food shortages. *β_N_* has been set to −0.1 meaning that the community primarily responds after a food shortage occurs (reactive). The transition between both regimes is gradual; *δ_N_* = 15. The parameters of vulnerability have been set so that only after a lasting food shortage a population response is initiated; *η_N_* = 15, with net growth rates reaching temporarily −0.0054 persons/month; *ζ_N_* = 4.5. *Cowgill* [1962] who has studied the practices (shifting agriculture) of present‐day farmers around Tikal has estimated that one household has enough labor potential to cultivate the land needed to support their family and also for a second household if needed. It is therefore reasonable that the value of *μ_N_* is somewhere between 1 and 2 and in the model it is set to 1.25. The water need per person per month, i.e., *γ_N_* set to 0.6, is chosen such that the carrying capacity of the land, without any adaptive measures being taken, matches the lowest population density estimate of 170 people per km^2^ provided by *Culbert* [1988].

The reservoir building and degradation rates have been calibrated to match the time period in which dam building has been observed in Tikal. The building rate is set to 0.01 mm per month per person, the degradation rate to 0.1 per month. Without maintenance, this implies that 70% of the storage is lost within a year if no maintenance takes place. Considering the monsoonal climate suffering from droughts and floods and the use of natural materials in order to create the structures, a high degradation rate due to gradual siltation of the system or occasional breaching seems likely [*Reddy and Behera*, 2009; *Baland et al*., 2007]. In the model, maintenance always takes place as it is linked to the presence of people, so the degradation rate is lower. The memory discount rate has been set to 0.3 per month, indicating that memory is mainly formed by the systems water status of the last 12 months. An overview of the parameters can be found in Table 1.

**Table 1:** Overview of Parameters Used in the Model

Parameter	Units^a^	Meaning	Figure 6	Figure 7	Figure 8
*Hydrology*					
*α_H_*	[mm month^– 1^]	*ET_crops_*	200		
*β_H_*	[mm month^– 1^]	*ET_native_*	200		
*γ_H_*	[month^– 1^]	*E_reservoir_*	0.04		
$\phi_{H}$	[mm]	field capacity	195		
*δ_H_*		runoff coefficient	10		
$\epsilon_{H}$		runoff coefficient	0.5		
*η_H_*		reservoir overflow	0.98		
*ζ_H_*		reservoir overflow	500		
*θ_H_*		yield response factor	1.25	1.25	1.1
*Demography*					
*α_N_*	[month^– 1^]	base rate	0.003		
*β_N_*		behavioral threshold	−0.1		
*γ_N_*	[mm month^– 1^ person^– 1^]	water demand per person	0.6		
*δ_N_*		adjustment speed	0.5		
$\epsilon_{N}$	[month^– 1]^	mortality rate	0.0012		
*ζ_N_*		response to V	4.5		
*η_N_*		response to V	15		
*θ_N_*	[month^– 1^]	birth rate	0.00095		
*μ_N_*		land to person ratio	1.25		
*Supply Management*					
*α_R_*	[mm month^– 1^ person^– 1^]	building rate per person	0, 0.01	0, 0.01	0, 0.01
*β_R_*	[person month^– 1^]	degradation rate per person	0.1		
Memory					
*α_M_*	[month^– 1^]	memory discount rate	0.3		
*Initial Values*					
*S*	[mm]	water storage	160		
*N*	[people km^– 2^]	population	20	80	20
*V*		vulnerability	0		
*M*		memory	0		
*R*	[mm]	reservoir capacity	0		
*P*	[mm month^– 1^]	precipitation	1800 mm annually on average		

a All parameters are per unit area.

### 4.2 Simulation

**Figure 6:**
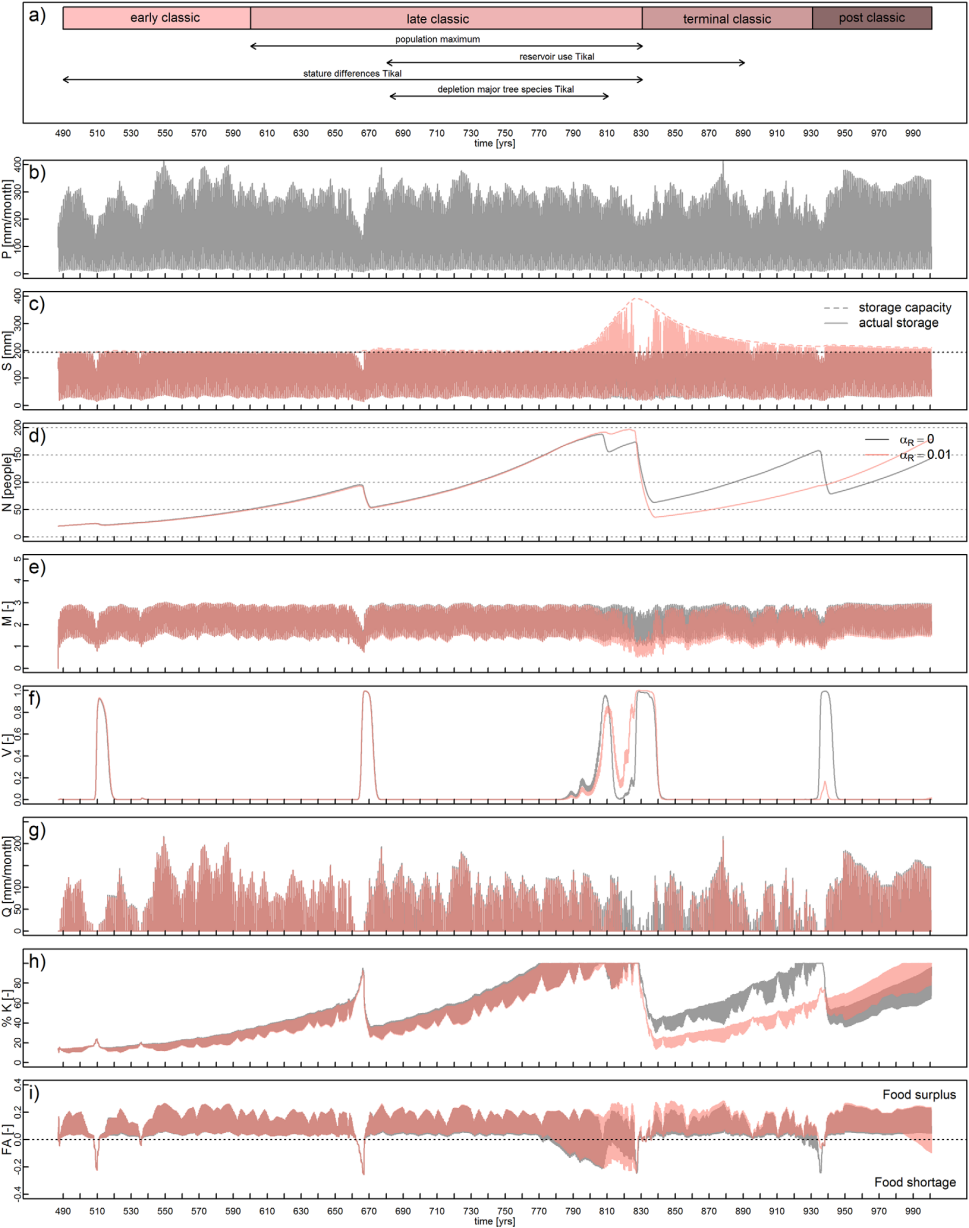
Scenario for a Maya center that does (pink) and does not (grey) build reservoirs. (a) Stylized facts based on the literature (see text for details). (b) Precipitation series based on *Medina‐Elizalde et al*. [2010]. (c) Actual storage and storage capacity. (d) Population dynamics. (e) Memory or social knowledge. (f) Vulnerability. (g) Runoff. (h) % of land use/carrying capacity i) Food availability.

Figure 6 shows the outcome of two simulations, one without reservoir construction (black) and one with the construction of reservoirs (pink). As indicated by *Medina‐Elizalde et al*. [2010] and shown in Figure 6b, periods of reduced rainfall that coincide with the disintegration of the Maya civilization occur around AD 806, 829, 842, 857, 895, 909, 921 and 935. A relatively water rich period occurred from AD 858 to 890. Relatively dry periods are furthermore centered around AD 510, 545 and 665. Figure 6c shows the total storage (soil moisture plus reservoir storage, solid line) in the catchment. Soil moisture storage, i.e., S < 195 mm, varies with the seasons and mean annual rainfall. As soon as the society builds reservoirs (long dashed pink line after the drought of 665), additional storage of water is possible and run‐off decreases Figure 6g). Construction of reservoirs however really takes off during AD 790 when the community again becomes vulnerable (Figure 6f) due to population pressure and decreasing food availability per person (respectively Figure 6h, representing the population in percent of the available carrying capacity, and Figure 6i, representing food availability). With additional reservoir storage the community is able to maintain its growth for a longer time period and reaches a higher population density (Figure 6d, pink line). However, when a dry period starts in AD 829, this higher density community is also more vulnerable and is hit harder by the drought than its nonreservoir counterpart. After the shock that resulted in major depopulation, the nonreservoir community that is impacted less is able to recover to almost preimpact population levels, after which it becomes again vulnerable around AD 935 as a consequence of its own success. During this time, the reservoir community also experiences a decrease in food availability but only a slight increase in vulnerability (see Figures 6i and 6f, respectively) due to a combination of lower population density and higher water storage. For comparison, Figure 6a shows the periods of maximum population reported in the literature [*Culbert*, 1988], reservoir use in Tikal [*Scarborough et al*., 2012], human stature changes possibly indicating food shortage [*Haviland*, 1967] and major tree species being depleted in Tikal forests [*Lentz and Hockaday*, 2009].

## 5 Discussion

This study pursues three goals, namely to present a simple, general model framework that is able to capture the major interactions between socio‐economic and hydrological systems, to apply it to the ancient Maya and to explore whether the drought hypothesis is consistent with the model framework. We will discuss the results starting with the last two goals. For the Maya case study, we presented two scenario's of the civilization's rise and fall (Figure 6). After the population experiences a maximum density during the Classic period, this expansion comes to an end around the drought of AD 830. The impact of the drought varies between the scenarios. An 80% decline of population is possible, but the drought impact can also be less severe with a faster recovery. According to the archaeological evidence both types of dynamics have been observed among Maya populations [*Culbert*, 1988; *Lucero*, 2002]. Rapid depopulation occurred for the center of Tikal, but also centers such as Calakmul experienced such a decline. On the other hand, occupation records indicate that despite the drought, populations continued to live in areas such as Belize and Barton Ramie and even flourished. This is also possible according to the results of the second scenario. The framework is thus capable of simulating plausible dynamics.

Another observation from the figure relates to the role of reservoirs. *Lucero* [2002] argues that, distinguishing between major, regional and minor centers, the major centers grew so large during the Classic Period because of their ability to capture and manage water efficiently through artificial reservoirs that allowed people to concentrate rather than scatter across the land. However, as the water source upon which society depended failed, the collapse experienced by these major cities was also more severe. As the only difference between the two scenarios presented here is the construction of reservoirs, they illustrate the dual nature of the dynamics in line with Lucero's observation. Additional storage brings benefits when the rains come, allowing the society to grow larger, but when the rains fail, the dependence of society on the additional water makes them more susceptible to population decline. While the outcome of the model supports the observed dynamics in a qualitative way, the actual population densities of the simulations with reservoirs do not match the high densities observed in a city like Tikal. Although, we have not included trade in the model as there is little to no evidence for large scale food trade, see section 3.3, it is likely that a major city center was dependent on food import from its directly surrounding lands to sustain the higher populations [*Santley et al*., 1986]. If a reconstruction with more spatial detail was of interest, these local food exchanges could be taken into account, for example by adding them to the food availability function of the model. The growth of the community is still limited by its maximum growth rate, even if it stores more water. Thus, the growth of the community to its higher carrying capacity level needs time. If a drought hits before the community has been able to exploit the full potential of the extra available water, the actual population density does not reach the maximum population density possible. While these dynamics could be changed in the model by allowing for more variation in growth rates to bring about higher population densities in a shorter period of time, more research would be needed on the type of processes driving this increase. For example, the migration literature discusses push and pull factors depending on whether a city is considered appealing [*Dorigo and Tobler*, 1983]. Maybe, reservoirs could have acted as a pull factor to the city, as is observed for contemporary cites as well [*Kallis*, 2010]. In this case, the role of networks through kinship, for example, would have to be explored as well, as networks facilitate information exchange and reduce the (social) costs of migration [*Choldin*, 1973; *Hunter et al*., 2015]. Furthermore, archaeological records indicate that for the center of Tikal, occupation never fully recovered in the Terminal Classic and Post Classic periods. The current simulation shows a gradual increase. While various processes could potentially have played a role as many possible explanations have been put forward for the Mayans' demographic trajectories, we like to stress two in this discussion. First, there is uncertainty associated with the results due to uncertain input data and second, as a result of the model simplicity, nonclimatic drivers are not considered. The precipitation series used is from an area northeast of Tikal. The series is based on isotope‐precipitation calibration (correlation coefficient r = 0.62) provided by *Medina‐Elizalde et al*. [2010]. In a review on methods used in paleoclimatology, *Douglas et al*. [2016] state that while isotopic records can give an indication of local climate variability, there are still large errors associated with such a calibration. With paleoclimatology and archeology progressing in extracting high resolution and ultimately spatially resolved climate data from the various Maya regions, it would be a valuable exercise to repeat the above simulations for various sites along the route of comparative socio‐hydrology. Using isotopic records from plant wax seems promising, as the method has reduced climatic and nonclimatic uncertainty making spatial comparison more feasible [*Douglas et al*., 2015, 2016]. A process that has likely played a role and that is not included in the model is warfare. *Kennett et al*. [2012], for example, propose a two stage drying collapse, where a drought around AD 660 triggered balkanization of polities, increased warfare and subsequently destabilization and disintegration between 800 and 900. This was followed by a more gradual population decline during the period between 1010 and 1100, a period which was also considered extremely dry. While the model does not explicitly include this feedback, it could be argued that it implicitly accounts for it through the mortality function. Most likely, warfare would have strengthened the dynamics observed in the simulation due to numerous interferences, such as relocation of labor from agriculture to warfare, increase in casualties and possibly migration [*Reuveny*, 2007; *Hunter et al*., 2015].

**Figure 7:**
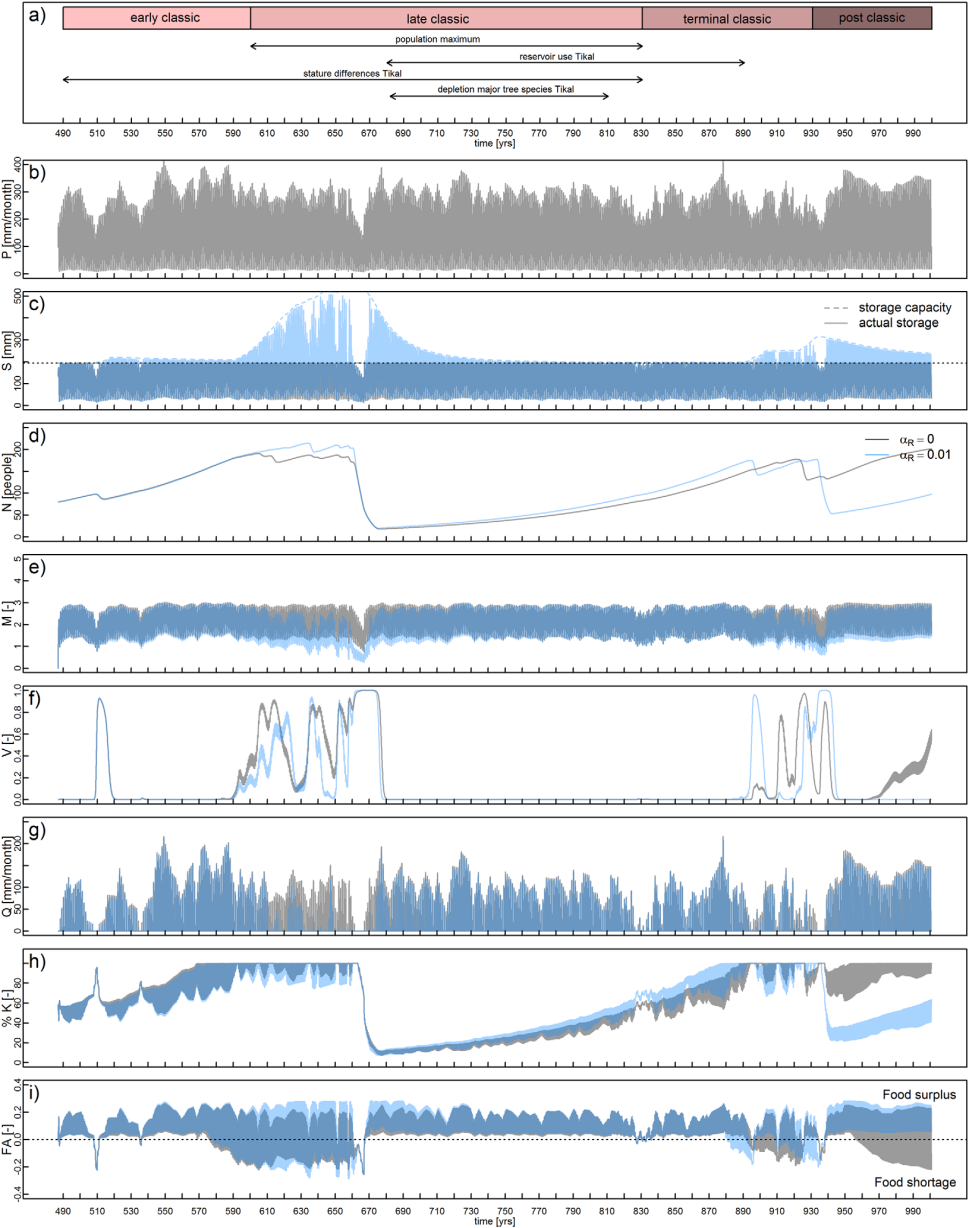
Scenario in which the initial population density is 80 instead of 20 people per km^2^ for a Maya center that does (blue) and does not (grey) build reservoirs. The variables presented in each plot are the same as in Figure 6.

**Figure 8:**
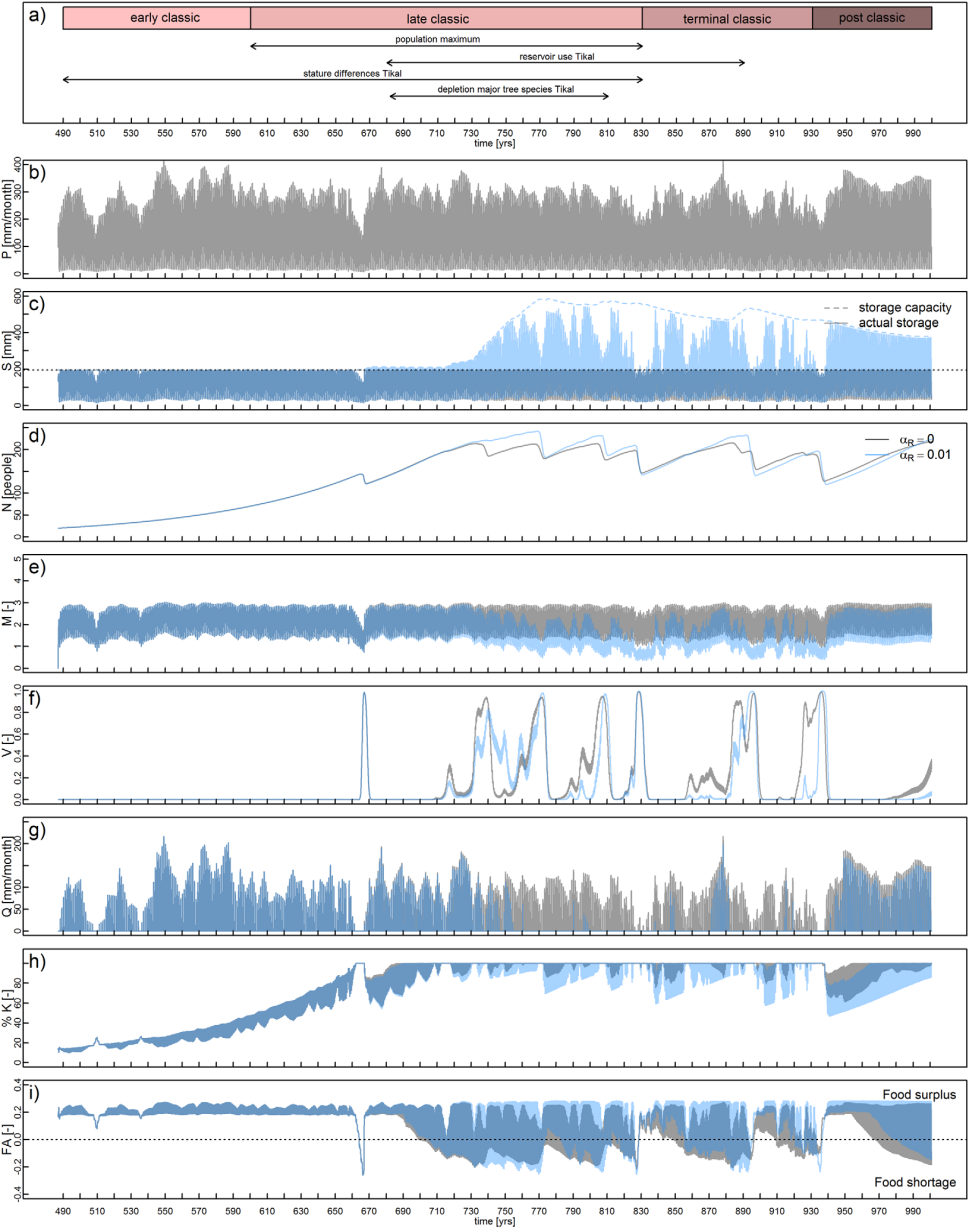
Scenario in which the yield response factor *θ_H_* is set to 1.1 rather than 1.25 (meaning that the crop is less sensitive to water stress) for a Maya center that does (blue) and does not (grey) build reservoirs. The variables presented in each plot are the same as in Figure 6.

The yield response factor also plays an important role. This parameter is kept constant over the years. In reality, crop yield will both depend on the hydrological and social dynamics. This is illustrated in two scenarios in Figures 7 and 8. In Figure 7, the initial population of 20 has been increased to 80 people per km^2^, which is still within the margin of 10–50% of maximum population density during the Early Classic reported by *Culbert* [1988] (see also section 3.2). The figure shows that a similar collapse could have occurred 50 years earlier or 60 to 100 years later, if the initial population had been larger, but with the same driving mechanisms. In Figure 8, the yield response factor is reduced from 1.25 to 1.1, meaning that the crop is less sensitive to water stress. Both the drought and the impact of the drought (determined by the yield response factor) are thus important for simulating an 80% population collapse. A general observation from the model is that having insights in local population densities, local land and water features, and its management are all relevant to understanding the societal dynamics of the Maya.

The model framework is intended to be simple and general, capturing the main feedbacks, so that it can be applied to various cases. One way forward with this model framework is to examine similar case studies in which water management, and specifically reservoirs, played an important role in the development of that society. One can think of this model as a framework to compare socio‐hydrological trajectories at different Maya sites, as well as those of other ancient societies such as the Khmer [*Buckley et al*., 2010]. The model could also be useful for a contemporary agricultural society. Depending on the case study, assumptions of self sufficiency, trade or additional water inflows could be relaxed. Examples that entail more local than global agricultural dynamics are small‐holder farmers in Africa or India. In the face of increasing population and climate variability, it is increasingly important that these communities are able to adapt and generate a stable income from their fields [*Vermeulen et al*., 2012]. In choosing the path perceived as most optimal, farmers need to continuously weigh both climatic and nonclimatic signals [*Bradshaw et al*., 2004]. Leaving the basic structure of S, P and V intact, one could replace the reservoir feedback by other adaptation strategies, e.g., actions that increase water use efficiency including crop choices. This would allow for the exploration of other socio‐hydrological feedbacks in isolation, but with the ultimate aim of working toward a model in which feedbacks are combined.

The rationale behind using the vulnerability concept as a base for the model framework is based on exactly the above idea. The strength of a framework that is general and capable of incorporating many types of feedbacks, whether they are purely technical or more intangible, is, however, also its weakness. In order for the term vulnerability to be comparable one needs to be consistent in its meaning or at least be clear on its definition. Based on the frequent usage of the term, the approach helps to conceptualize, organize and discuss the various actions and responses within complex human‐environmental systems [*Turner et al*., 2003; *Eakin and Luers*, 2006]. Thus, trying to quantify vulnerability explicitly might aid in defining these feedbacks further, in order to recognize the feedbacks and specify their timing, locations and strengths in order to obtain qualitative plausible behavior [*Kumar*, 2015].

The model simulates two kinds of population dynamics: a subsistence equilibrium as is inherent to Malthus theory, and a form of overshoot behavior, possibly resulting in a collapse, which is discussed in the limits to growth literature [*Meadows et al*., 1972] and often associated with the demise of some ancient societies (e.g., the Easter Island population [*Brander and Taylor*, 1998], the Indus civilization, the Hohokam society [*Pande et al*., 2013], the Khmer empire [*Buckley et al*., 2010]). For present‐day industrial societies this behavior is not usually observed, indicating that the model is less suitable for these. However, contrary to the common notion that an industrial society is characterized by a post‐Malthusian regime, one could also argue that we live in a Malthusian world that has experienced high rates of technological progress and that we have not yet reached the limit [*Cohen*, 1995]. As is clear from the economic literature, an important driver of economic growth is technological change [*Solow*, 1957; *Romer*, 1989] among more recent proposed determinants such as geography [*Haber*, 2012; *Sachs and Warner*, 1995] and institutions [*North*, 1990]. Neoclassical growth theory suggests that as long as technological change outweighs the decrease in marginal returns coming from labor, capital, and (limited) natural resources, the final outcome is growth and not stagnation or collapse. Within a socio‐hydrological system striving for water security, a similar analogy can be made: as long as technological change, and management and market responses keep in pace with the increase in total demand, Malthus' limit has not been reached. Measures of increasing supply or reducing the per capita demand are then aimed at extending the (local) carrying capacity of the system. In order to assess the effect of a measure on the hydrological system, the notion of purely technological change as represented in macro economic models is probably too narrow. One can picture a society responding to (expected) signals of its environment, e.g., water scarcity, and acting accordingly. The model framework as it is now, has isolated one feedback, i.e., reservoir building, but one can imagine that collecting all the types of feedbacks and knowing the strength of these feedbacks, alias our 'adaptive capacity', collectively pushes the carrying capacity upward. Hence, realizing that the adaptive capacity element has generally been winning in the past decades, the model framework as presented here could still be useful for present‐day societies.

While the model simulates plausible dynamics, the outcome is sensitive to its parameters and inputs, making predictions difficult. Further work could involve sensitivity analyes to evaluate under what circumstances dominant outcomes are obtained. *Viglione et al*. [2014], for example, analyzed the role of memory, risk‐taking attitude and trust in the development of a flood prone society and found that path dependency of the society, where the sequence of flooding controlled the development of the community resulting in either prosperity or demise. Using equilibrium solutions to determine the long‐run behavior of a model is a technique often used in macro‐economic modeling and has recently been applied to socio‐hydrological modeling to provide insight in to the trade‐off between flood defense investment and economic growth [*Grames et al*., 2015]. Similarly, the proposed model could be used in a probabilistic mode to provide insight into the paths of socio‐hydrological systems. Validating the model, however, still remains a challenge as long as there is little knowledge of the different strengths of feedbacks that drive the system. Building up a rich data set will help in testing the model more thoroughly [*Cox*, 2015].

## 6 Conclusion

The analyses indicate that it is possible to simulate plausible socio‐hydrological drought processes using a stylized model consisting of five differential equations. The simple stylized formulation gives sufficiently rich dynamics to simulate the depopulation of the city of Tikal around A.D. 830 and confirms the possibility of other possible socio‐hydrological pathways, i.e., a temporal alleviation after the initial shock followed by a collapse later in history, as is also observed in the archaeological data. The final outcome is an interplay between the exposure, sensitivity and adaptive capacity of the system. In all simulations, reservoirs have a twofold effect allowing the society to maintain and increase their wealth for longer, but when reservoirs dry up the drop in population is bigger. Future work may focus on sensitivity analyses to more comprehensively map the feedbacks between the social and hydrological system components.
